# Utilization of denture adhesives and the factors associated with its use: a cross-sectional survey

**DOI:** 10.1186/s12903-020-01177-5

**Published:** 2020-07-08

**Authors:** Tun Min Bo, Yohei Hama, Norihisa Akiba, Shunsuke Minakuchi

**Affiliations:** Department of Gerodontology and Oral Rehabilitation, Graduate School of Medical and Dental Sciences, Tokyo Medical and Dental University, Tokyo, Japan

**Keywords:** Denture adhesives, Complete denture, Partial denture, Last dental visit, Smoking, Elderly, Edentulous

## Abstract

**Background:**

An increase in the number of elderly edentulous patients likely leads to a greater demand for dentures and denture adhesives. As denture adhesives have both positive and negative features, dentists need to know the types of denture adhesive users to be able to provide instruction to denture wearers on how to use denture adhesives effectively. This study aims to examine the utilization of denture adhesives and associated factors.

**Methods:**

Seven closed-ended questionnaires were developed for a web-based survey. After that, Fisher’s exact tests were performed to determine the difference in the denture adhesive usage rate by gender, type of denture, last dental visit, and smoking status. A multivariate-adjusted logistic regression analysis was performed with denture adhesive use or non-use as the dependent variable and the other items as independent variables. Next, Fisher’s exact tests and a multivariate-adjusted logistic regression analysis were performed with the type of denture adhesives as the dependent variable in the same way. Statistical analyses were performed for all denture wearers, complete denture wearers, and partial denture wearers. The level of statistical significance was set at *p* = 0.05.

**Results:**

A total of 1470 denture wearers in Japan participated in this study. Of these, 318 used denture adhesives, while 212 (66.7%) used cream; 74 (23.3%) used home liner; 25 (7.9%) used powder; four (1.3%) used sheets; and three (0.9%) used several types. The Fisher’s exact tests revealed that the ratios of using denture adhesives were significantly higher for complete denture wearers (*p* < 0.001), last dental visit over 1 year (*p* = 0.005), and smokers (*p* = 0.005). For partial denture wearers, the ratio was significantly higher in smokers (*p* = 0.262). The multivariate adjusted logistic regression revealed that denture adhesive use or non-use were significantly associated with the type of denture and smoking status in all denture wearers, and just smoking status in partial denture wearers. There were no significant results about the type of denture adhesive selection.

**Conclusions:**

From all denture wearers, complete denture wearers and smokers are more likely to use denture adhesives. In addition, smokers significantly use denture adhesives if they are partial denture wearers.

## Background

The proportion of the older population in Japan has been increasing in the past years [1]. The Japanese government projected that the proportion of older persons above 65 years of age would further increase to 38.4% by 2065 [1]. With the ever-increasing elderly population, there will inevitably be greater challenges for the delivery of oral health care. Despite progress in dentistry, tooth loss that comes with old age is still a reality [2, 3]. There is an expectation of complete edentulism in 2.9 to 58% of the adult population internationally [4, 5]. With the increase in the number of elderly edentulous patients, there is likely to be a greater demand for complete or partial dentures. Some of the main problems posed by complete denture wearers involve retention, stability, and function. These are important indicators in estimating masticatory ability and oral health-related quality of life [6].

Furthermore, functional disturbances, as well as psychological problems, were found in complete denture wearers as well [7]. More than 40% of complete denture wearers complained of mastication discomfort or pain and looseness of their dentures [8]. There were also some problems encountered in partial dentures. The most frequently encountered complication in partial removable dentures involved the loss of retention – causing dissatisfaction of patients related to chewing ability [9]. Needless to say, denture wearers with denture problems should visit their respective dental clinic to remedy such problems. However, some elderly people have difficulty getting professional dental treatment because of their physical disabilities. A previous study reported that a majority (96%) of 125 homebound elderly in New York City had never been visited by dental professionals at home since they became homebound, and 59.4% of denture wearers were dissatisfied with their current dentures [10].

In order to solve these common problems, denture adhesives are being utilized widely by denture wearers [11]. Denture adhesives have attracted more attention since guidelines for the use of denture adhesive was published in 2019 by the Oral Health Foundation [12]. Program directors of undergraduate complete dentures curriculum in dental schools in the United States agreed that denture adhesives are used to improve the overall performance of complete dentures [13]. Moreover, 61.5% of general dental practitioners and 49.0% of specialist prosthodontists in Athens, Greece, recommended the use of denture adhesives [14]. Such use also aids in psychological satisfaction, thus improving the quality of life [15]. Denture adhesives improved well-fitting denture stability [16], mastication [17], patient satisfaction [18], mandibular movement during chewing [18], and maximum bite force until denture dislodgement [19, 20]. Experts also use denture adhesives in the clinical process when making dentures, particularly in study base fixation, bite registration, and improvement of the accuracy of denture try-ins [21]. The use of denture adhesives significantly increases the force required to displace mandibular free-end saddle partial dentures [22].

However, some previous studies reported that denture adhesives also have some negative aspects [11]. Program directors of dental schools in the United States agreed that denture adhesive can contribute to residual ridge resorption [13]. Moreover, denture adhesives could promote the development of oral diseases/conditions such as denture stomatitis, candidiasis, and oral flora imbalance [13]. Although recent studies reported that short-term use of denture adhesives (15 days or 2 months) did not negatively affect oral microorganisms [23, 24], the influence of long-term use is still unknown.

Since denture adhesives may have not only positive but also negative aspects, they must be used in an appropriate manner with accurate knowledge. Thus, dentists need to know the kinds of denture adhesive users to instruct them on how to use denture adhesives properly and effectively.

However, previous studies were only small surveys with under 150 participants [25] and medium scale surveys with 449 participants [26]. These studies did not obtain information on factors associated with the use of denture adhesives. To the best of our knowledge, studies that investigated factors associated with the use of denture adhesives are limited [27]. Thus, we performed a large-scale survey with numerous participants using a web-system to investigate denture adhesives users in detail. This study aimed to survey the utilization of denture adhesives of denture wearers and to determine the factors associated with the use of such denture adhesives.

## Methods

### The characteristics of participants

We used the services of a web-survey company (OGIS-RI Co., Ltd.) to develop a web-based survey system for denture adhesive users in Japan. The company provided access to 92,747 people who previously registered in the web-based survey system and were 55 years of age or older. We selected the participants for this study from respondents of the web system. Inclusion criteria were denture wearers without missing answers.

### Web-questionnaire

We developed an electronic closed-ended questionnaire in Japanese, consisting of seven questions. The following information was obtained: 1) *gender*, 2) *age*, 3–1) *denture wearer or not*, 3–2) *the type of denture* (complete denture or partial denture), 3–3) *denture adhesive user or not*, 3–4) *the type of denture adhesive* (cream, home liner, powder, sheet, or several-types user) 4) *last dental visit under 1 year or more than 1 year*, and 5) *smoking or not*. Question numbers 3–2, 3–3, and 3–4 were asked only to denture wearers who answered “yes” to Question 3–1 by the web-survey system. An English version of the questionnaire is available as a supplement to this paper.

### Statistical analysis

Information on the utilization of denture adhesives by denture wearers was first presented. After that, Fisher’s exact tests were performed to determine the difference in denture adhesive usage rate by gender, type of denture, last dental visit, and smoking. Moreover, multivariate adjusted logistic regression analysis was performed with denture adhesive use or non-use as the dependent variable and the other items as independent variables. These analyses were performed again for complete and partial denture wearers, respectively. Next, we analyzed the type of denture adhesive. Cream, powder, and sheet were categorized into narrowly-defined denture adhesive, and the home liner was categorized into the home liner. Several-types users were removed from the analysis. Fisher’s exact tests and multivariate adjusted logistic regression analysis were performed with the type of denture adhesive as the dependent variable in the same way. The level of statistical significance was set at *p* = 0.05. Data were analyzed using JMP version 8.0 (SAS Institute, Cary, NC, USA).

## Results

### Characteristics of participants

Out of 92,747 persons asked to answer the survey, only 23,424 replied using the web-survey system. Among these replies, only 5935 had valid answers without any missing values, and the final response rate was 6.4%. This study obtained and analyzed 1470 denture wearers. The characteristics of these participants are shown in Table 1.

**Table 1:** Characteristics of the participants

		All denture wearers		Complete denture wearers		Partial denture wearers	
N		1470		386		1084	
Age	Median Year (Min.-Max.)	70 (55–89)		71 (55–89)		70 (55–86)	
Gender							
Male		1153	(78.4%)	331	(85.7%)	822	(75.8%)
Female		317	(21.6%)	55	(14.3%)	262	(24.2%)
Last dental visit							
under 1 year		1053	(71.6%)	202	(52.3%)	851	(78.5%)
over 1 year		417	(28.4%)	184	(47.7%)	233	(21.5%)
Smoking							
non-smoker		1189	(80.9%)	288	(74.6%)	901	(83.1%)
Smoker		281	(19.1%)	98	(25.4%)	183	(16.9%)

### Denture adhesive utilization

The survey results on denture adhesive use and the corresponding type of denture adhesives utilized are shown in Table 2.

**Table 2:** Utilization survey of denture adhesives

	All denture wearers		Complete denture wearers		Partial denture wearers	
Denture adhesive						
user	318	(21.6%)	184	(47.7%)	134	(12.4%)
non-user	1152	(78.4%)	202	(52.3%)	950	(87.6%)
Type of denture adhesive						
cream	212	(66.7%)	122	(66.3%)	90	(67.2%)
home liner	74	(23.3%)	43	(23.4%)	31	(23.1%)
powder	25	(7.9%)	14	(7.6%)	11	(8.2%)
sheet	4	(1.3%)	3	(1.6%)	1	(0.7%)
Several-types user	3	(0.9%)	2	(1.1%)	1	(0.7%)

### Related factors for denture adhesive use

Fisher’s exact tests revealed that for all denture wearers, the ratios of using denture adhesives were significantly higher for complete denture wearers (*p* < 0.001), last dental visit over 1 year (*p* < 0.001), and smokers (*p* = 0.005). On the other hand, for partial denture wearers, the ratio was significantly higher in smokers (*p* = 0.262). The complete results of the usage ratio are shown in Table 3.

**Table 3:** The usage ratio of denture adhesives

	All denture wearers		Complete denture wearers		Partial denture wearers	
		*p*-Values		*p*-Values		*p*-Values
Gender						
Male	22.5%	0.14	49.6%	0.08	11.6%	0.16
Female	18.6%		36.4%		14.9%	
Last dental visit						
under 1 year	**18.6%**	**< 0.001**	49.5%	0.48	11.6%	0.18
over 1 year	**28.5%**		45.7%		15.0%	
Smoking						
non-smoker	**19.8%**	**< 0.001**	46.2%	0.35	**11.3%**	**0.026**
smokers	**29.5%**		52.0%		**17.5%**	
Type of denture						
partial denture	**12.4%**	**< 0.001**				
complete denture	**47.7%**					

Bold faces are significant variables in Fisher’s exact tests (*p* < 0.05)

Multivariate-adjusted logistic regression revealed that denture adhesive use or non-use were significantly associated with the type of denture and smoking in all denture wearers, and smoking in partial denture wearers. These results are shown in Table 4.

**Table 4:** Multivariate adjusted logistic regression analyses using denture adhesive use or not as dependent variables

Independent Variables	All denture wearer		Complete denture wearer		Partial denture wearer	
	OR (95%CI)	*p*-Values	OR (95%CI)	*p*-Values	OR (95%CI)	*p*-Values
Age	0.99 (0.97–1.02)	0.58	0.99 (0.95–1.02)	0.45	1.00 (0.96–1.03)	0.89
Gender						
male	1.00		1.00		1.00	
female	1.03 (0.73–1.47)	0.84	0.54 (0.30–1.00)	0.05	1.45 (0.959–2.21)	0.08
Last dental visit						
under 1 year	1.00		1.00		1.00	
over 1 year	1.03 (0.77–1.39)	0.85	0.81 (0.540–1.22)	0.31	1.29 (0.85–1.97)	0.23
Smoking						
non-smoker	1.00		1.00		1.00	
smoker	**1.44 (1.04–2.00)**	**0.03**	1.22 (0.761–1.95)	0.41	**1.72 (1.09–2.69)**	**0.02**
Type of denture						
partial denture	1.00					
complete denture	**6.36 (4.78–8.47)**	**< 0.001**				

OR (95%CI): Odds ratio (95% confidence interval)

Bold faces are significant variables (*p* < 0.05)

### Related factors for the type of denture adhesive selection

Fisher’s exact tests and multivariate-adjusted logistic regression with the type of denture adhesive as the dependent variable did not indicate significant results, as shown in Tables 5 and 6.

**Table 5:** The usage ratio of narrowly-defined denture adhesive (cream, powder, and sheet)

	All denture wearer		Complete denture wearer		Partial denture wearer	
		*p*-Values		*p*-Values		*p*-Values
Gender						
Male	75.9%	0.73	75.9%	0.79	75.8%	0.82
Female	79.3%		80.0%		79.0%	
Last dental visit						
under 1 year	77.7%	0.58	78.8%	0.48	76.5%	1.00
over 1 year	74.6%		73.5%		77.1%	
Smoking						
non-smoker	77.9%	0.36	76.7%	0.85	79.4%	0.23
smoker	72.5%		75.5%		67.7%	
Type of denture						
partial denture	76.7%	1.00				
complete denture	76.4%

**Table 6:** Multivariate adjusted logistic regression analyses using narrowly-defined denture adhesive (cream, powder, and sheet) use or not as dependent variables

Independent Variables	All denture wearer		Complete denture wearer		Partial denture wearer	
	OR (95%CI)	*p*-Values	OR (95%CI)	*p*-Values	OR (95%CI)	*p*-Values
Age	0.97 (0.92–1.01)	0.16	0.97 (0.91–1.03)	0.36	0.96 (0.89–1.03)	0.28
Gender						
male	1.00		1.00		1.00	
female	1.07 (0.52–2.24)	0.85	1.09 (0.33–3.56)	0.36	0.98 (0.38–2.57)	0.97
Last dental visit						
under 1 year	1.00		1.00		1.00	
over 1 year	0.81 (0.47–1.41)	0.45	0.72 (0.36–1.45)	0.36	0.98 (0.39–2.50)	0.98
Smoking						
non-smoker	1.00		1.00		1.00	
smoker	0.72 (0.40–1.31)	0.28	0.90 (0.41–1.97)	0.80	0.52 (0.20–1.34)	0.17
Type of denture						
partial denture	1.00					
complete denture	1.12 (0.64–1.97)	0.68				

OR (95%CI): Odds ratio (95% confidence interval)

## Discussion

This study uses the largest survey among existing studies relating to denture adhesives using a web-based survey system. A substantial proportion of denture wearers, which was 1470 persons, were investigated for the use of denture adhesives. As a result, we revealed the utilization of denture adhesives and the factors related to the use or non-use of denture adhesives.

The number of participants greatly differed by gender. There were 1153 male and 317 female participants. This gender gap was because participants could freely participate, and no rule on gender was specifically determined for this study. Previous studies showed that general and oral health literacy and their related factors differed between genders [28, 29]. Therefore, in the same way, the related factor to denture adhesive use or non-use is possibly different between genders. Thus, we have to recognize that the gender gap may affect the results of this study. As this study was a web-based survey, it was expected before starting the survey that there would be a limitation regarding computer-use for older age groups. Therefore, the majority of the participants may come from the younger age groups. However, the group of participants was still widely distributed in this study. The median age was 70, while the maximum and minimum age were 89 and 55, respectively. Therefore, an analysis of a wide age group was possible in this particular study.

There were 318 (21.6%) out of 1470 denture adhesives users in this study, as shown in Table 2. Coates reported that 6.9% of participants in his study used denture adhesive [25]. Meanwhile, Polyzois reported that 26 and 20% of Greek and Dutch participants, respectively, used denture adhesives [26]. It is not surprising that the usage rate of denture adhesives differs 6.9–26% by region or time. Regarding the types of adhesives used, as shown in Table 2, cream-type users were the majority with home liner users coming in second. These usage rates of the type of denture adhesives showed the same trend in complete and partial denture wearers.

To investigate the difference in usage rate of denture adhesives by gender, the type of denture, last dental visit, and smoking, Fisher’s exact probability tests were performed (Table 3). The usage rate of denture adhesives differs greatly between partial denture wearers (12.4%) and complete denture wearers (47.7%). Moreover, multivariate adjusted logistic regression analysis using age, gender, the type of denture, last dental visit, and smoking, revealed that complete denture wearing and smoking were significant factors (Table 4). This means that complete denture wearers used denture adhesives about 6.36 times more than partial denture wearers, and smokers used them about 1.44 times more than non-smokers. Denture type had a particularly powerful influence. One of the main effects of denture adhesive is the improvement of retention [16]. Needless to say, the retentive mechanism is different between complete and partial dentures with retention mechanisms such as clasps. We considered the related factors of using a denture adhesive to be different between complete and partial denture wearers. We then performed analyses of complete and partial denture wearers separately in further statistical analyses.

In complete denture wearers, to investigate the difference in usage rate of denture adhesives by gender, type of denture, last dental visit, and smoking, Fisher’s exact probability tests were performed (Table 3). Moreover, multivariate adjusted logistic regression analysis was also performed (Table 4). Though both tests do not show statistically significant results, the logistic regression showed that a 95% confidence interval of the odds ratio of female to male was 0.30–1.00. Simply stated, this indicates that female use of denture adhesives is 0.30 to 1.00 times that of male use with a probability of 95%. Although there was not enough evidence, males have a probability of using denture adhesive more, which might be caused by the confounding factor of some male backgrounds. To reveal the various related factors in using denture adhesives in complete denture wearers, further studies are needed that investigate the denture quality and frequency of denture use reported by previous studies [27].

Next, we performed statistical analyses only in partial denture wearers. Fisher’s exact probability tests revealed that smokers used denture adhesive more (Table 3). Furthermore, multivariate adjusted logistic regression analysis revealed that smoking was a statistically significant factor. Therefore, smokers used denture adhesive about 1.72 times more than non-smoker (Table 4). Smoking can cause reduced salivary flow or dry mouth -- called xerostomia [30]. This is one of the reasons why smokers who are partial denture wearers use denture adhesive. Saliva plays an important role for dentures. The stimulated salivary flow rate was significantly related to masticatory performance in Eichner group C denture wearers [31].

Moreover, we analyzed related factors for the type of denture adhesive selected. Unfortunately, we did not find significant results, as shown in Tables 5 and 6. Further studies are needed to investigate this subject.

We recognize some limitations in the scope of our study. First, it was sampling bias. This study used a web-based system, and therefore all participants were limited to those who could access the internet. Moreover, the response rate of this study was low because of missing answers. As a result, participants were considered to be independent elderly who had high information literacy skills and did not have severe problems related to activities of daily living. Second, a web-survey system can collect subjective answers of the questionnaire by participants, but cannot collect objective data measured by an examiner, such as objective masticatory performance and denture condition. Taking these limitations into consideration, this is the first study that investigates the utilization of denture adhesive in 1470 denture wearers.

We partially revealed related factors to denture adhesive use. That is to say, in all denture wearers, smokers, and complete denture wearers use more denture adhesive. Moreover, smokers used more denture adhesive if they were partial denture wearers. Therefore, dentists have to see these patients more carefully in consideration of the possibility of the use of denture adhesives and provide appropriate guidance on the effective and appropriate use of denture adhesives. Further studies should be performed to explain the related factors of denture adhesive use in complete denture wearers and the related factors of the type of denture adhesive selection. Moreover, the related factors of denture adhesive use should be examined in greater detail to combine web-survey data with data that was directly examined.

## Conclusions

The usage rate of denture adhesives in this web-based survey was 21.6% in all denture wearers, 47.7% in complete denture wearers, and 12.4% in partial denture wearers. The usage rates by the type of denture adhesives in descending order were cream, home liner, powder, sheet, and several-types user. In all denture wearers, complete denture wearers and smokers used significantly more denture adhesives. Meanwhile, in partial denture wearers, smokers used significantly more denture adhesives.

## Supplementary information

**Additional file 1.**

## Data Availability

All data files are available from the corresponding author upon request.
