# Supplementary material for: Utilization of denture adhesives and the factors associated with its use: a cross-sectional survey
Source: BMC Oral Health. 2020 Jul 8;20:194. doi: 10.1186/s12903-020-01177-5 (PMC7346652; doi:10.1186/s12903-020-01177-5)
Supplement: Supplementary file 1 — Additional file 1. [file 12903_2020_1177_MOESM1_ESM.docx]

Web-Based Questionnaire (original version: In Japanese)
Q1. Which is your gender?

a. Man b. Woman

Q2. How old are you?

Q3-1. Do you use removable dentures?

a. Yes b. No

Q3-2. What type of dentures are your dentures?

a. Complete denture b. Partial denture c. Both

Q3-3. Do you use denture adhesives?

a. Yes b. No

Q3-4. What type of denture adhesive do you use?

a. Cream b. Home liner c. Powder d. Sheet e. Several-types

Q4. When did you last visit the dental clinic?

1. Within 1-year b. More than 1-year ago

Q5. Are you a smoker?

a. Yes b. No
